# *In-Vitro α*-amylase, *α*-glucosidase Inhibitory Activities and *In-Vivo* Anti-Hyperglycemic Potential of Different Dosage Forms of Guduchi (*Tinospora Cordifolia* [Willd.] Miers) Prepared With Ayurvedic Bhavana Process

**DOI:** 10.3389/fphar.2021.642300

**Published:** 2021-05-10

**Authors:** Rohit Sharma, Rajesh Bolleddu, Jayanta K. Maji, Galib Ruknuddin, Pradeep K. Prajapati

**Affiliations:** ^1^ Department of Ras Shastra and Bhaishajya Kalpana, Faculty of Ayurveda, Institute of Medical Sciences, Banaras Hindu University, Varanasi, India; ^2^ Central Ayurveda Research Institute, CCRAS, Ministry of AYUSH, Government of India, Kolkata, West Bengal, India; ^3^ Society for Research and Initiatives for Sustainable Technologies and Institutions (SRISTI), Ahmedabad, India; ^4^ Department of Ras Shastra and Bhaishajya Kalpana, All India Institute of Ayurveda, University of Delhi, New Delhi, India

**Keywords:** tinospora cordifolia, guduchi, bhavana, *α*-amylase, *α*-glucosidase, anti-hyperglycemic, hypoglycemic

## Abstract

*Guduchi* (*Tinospora cordifolia* [Willd.] Miers) is a flagship rejuvenating herb of Ayurveda with reported anti-diabetic potential. In the present study, different dosage forms of *Guduchi* stem (growing on neem tree) were developed by adopting Ayurvedic pharmaceutical process of *Bhavana* (levigation). *Guduchi Churna* (GC) was subjected to 07 times *Bhavana* separately with its own extracted juice, decoction and potable water, and dosage forms namely *Svarasa Bhavita Guduchi Churna* (SBGC), *Kwatha Bhavita Guduchi Churna* (KBGC), and *Jala Bhavita Guduchi Churna* (JBGC) were prepared. The present study was aimed to evaluate the role of *Bhavana* on the potentiation of therapeutic properties of *Guduchi*. Sequential solvent extracts (5, 10, 15 and 25%) of GC, SBGC, KBGC and JBGC were prepared in different solvents [phosphate buffer, hexane, dichloromethane (DCM), chloroform] and screened for the *α*-amylase and *α*-glucosidase inhibitory activity. The results revealed that phosphate buffer and DCM extracts of SBGC exhibited strong *α*-amylase inhibitory potential (>80% inhibition at 25% concentration) followed by KBGC, JBGC and GC with reference to the standard acarbose. In *α*-glucosidase inhibitory activity, maximum inhibition was observed in DCM and chloroform extracts of SBGC (>85% inhibition at 25% concentration), followed by KBGC (>80% inhibition at 25% concentration), JBGC and GC. *In vivo* anti-hyperglycemic studies were carried out by oral glucose tolerance test in Swiss albino mice. Test drugs (JBGC, KBGC, SBGC) treated groups showed marginal decrease of blood glucose levels in normo glycemic mice. However, the blood glucose level in test drug JBGC, KBGC and SBGC treated groups was still within normal range in overnight fasted mice. In oral glucose tolerance test, among all dosage forms SBGC (51.08%) produced pronounced anti-hyperglycemic effect followed by KBGC (42.57%) at a dose of 520 mg/kg. The GC, JBGC, KBGC and SBGC samples were also standardized using berberine (a well established anti-diabetic compound) as a marker compound by HPTLC fingerprint analysis. Findings of the present study indicate that SBGC and KBGC can be used in the treatment of diabetes mellitus and gives supporting evidence to Ayurvedic claims that the *Bhavana* process has pharmaceutico-therapeutic significance in Ayurvedic drug development.

## Introduction

Diabetes mellitus (DM) is a chronic metabolic disorder caused by a failure in insulin production or a decrease in insulin sensitivity and function, affecting the lipid and carbohydrate metabolism. Hyperglycemia, an inevitable ramification of diabetes, is linked with several deleterious effects associated with this disease (Sharma et al., 2015a). DM is exponentially growing worldwide and leading cause of morbidity and mortality in low- and middle-income countries (Olowoyo et al., 2018). Based on current report of International Diabetes Federation (IDF) the global number of diabetic people (aged 18–99 years) will increase to 693 million by 2045 (Cho et al., 2018). Most of the conventional anti-diabetic drugs are reported to have side effects in long term use and have certain limitations, and options from natural products especially herbal medicine are being searched to meet the need (Sharma et al., 2015a).

*Guduchi* (*Tinospora cordifolia* Willd. Miers.; Family: Menispermaceae) is a renowned, deciduous, extensively spreading, climbing, herbaceous vine, distributed throughout the tropical Indian subcontinent and China, ascending to an altitude of 300 m. Mainly three *Tinospora* species are available in India, namely *T. cordifolia, T. malabarica/T. sinensis* and *T. crispa*. Though these species are closely related with their morphology and chemical properties, amongst them, the level of berberine (a well established anti-diabetic compound) is reported higher in *T. cordifolia*. The name - *T. cordifolia* is ascribed to the Ayurvedic plant *Guduchi* in Ayurvedic Pharmacopoeia of India and the traditional practitioners also consider *T. cordifolia* as the genuine source for *Guduchi* (Srinivasan et al., 2008; Nidhi et al., 2013). *Guduchi* is traditionally used in Indian Ayurveda medicine to treat wide range of diseases such as fever, diabetes, urinary disorders, anemia, jaundice, asthma, stress, allergy, skin disorders, arthritis, liver disorders etc., (Saha and Ghosh, 2012; Sharma et al., 2014a). Several reports substantiate its high anti-diabetic potential (Sharma et al., 2015a). Time by time its different traditional formulations like *Churna* (fine powder), *Kwatha* (decoction), *Satva* (sedimented starchy aqueous extract) and *Ghana* (solidified aqueous extract) were developed according to its need, and their anti-diabetic potential is evident from several studies (Sharma et al., 2013a; Sharma et al., 2013b; Sharma et al., 2015b; Gade, 2017).

*Bhavana* (levigation or wet-grinding of powdered drugs with juice/decoction/solution of plant, animal or mineral origin) is a unique traditional method of transformation of raw material/substances and process of herbal drug manufacturing, affecting the physicochemical and biological properties of a dosage form. *Bhavana* is one of the most commonly carried out pharmaceutical process in Ayurveda, having multi-dimensional pharmaceutico-therapeutic implications and various popular traditional herbal or herbo-mineral formulations are being prepared by this process. *Bhavana* process is claimed to make quicker and augmented action with possible reduction in therapeutic dose of the drug under process (Sharma and Prajapati, 2015; Sharma et al., 2017). *Churnakriya* is a type of *Bhavana,* wherein *Bhavana* of juice/decoction of the same drug is given usually with the motto of augmentation of properties of the drug being levigated and, hence, to potentiate the therapeutic action (Acharya, 2004). By adopting *Churnakriya* method of *Bhavana*, the potency of single herbs like *Guduchi* can be improved.

To the best of our knowledge, to date, there is no scientific evidence on the inhibitory effects of any dosage form or formulation of *Guduchi* on carbohydrate hydrolyzing enzymes. Also, no reports are available exploring anti-hyperglycemic potential of *Bhavita* dosage form of *Guduchi*. Considering all these, the present study was planned to validate the classical uses of *Guduchi* and its preparations in diabetes and understand the role of *Bhavana* in herbal drug potentiation, especially whether *Bhavana* process could augment the therapeutic potential of *Guduchi Churna* (GC) to manage glycemic levels. GC was subjected to 07 times *Bhavana* separately with its own extracted juice, decoction and potable water, and the dosage forms namely *Svarasa Bhavita Guduchi Churna* (SBGC), *Kwatha Bhavita Guduchi Churna* (KBGC), and *Jala Bhavita Guduchi Churna* (JBGC) were prepared. The third trial preparation JBGC was prepared (by subjecting *Bhavana* to GC with potable water) to obtain the same particle size reduction as in first two groups and to understand the effect of *Bhavana*. These preparations were comparatively evaluated for *in-vitro α*-amylase, *α*-glucosidase inhibitory activities and *in-vivo* anti-hyperglycemic potential with High Performance Thin Layer Chromatography (HPTLC) profiling.

## Materials and Methods

### Preparation of Test Formulations

JBGC, KBGC and SBGC samples were prepared by adopting classical Ayurvedic pharmaceutical guidelines of *Bhavana* process (Sharma and Prajapati, 2015; Sharma et al., 2017) in the department of Rasa Shastra and Bhaishajya Kalpana, Institute for Postgraduate Teaching and Research in Ayurveda, Gujarat Ayurved University, Jamnagar, Gujarat, India.

For preparation of *Guduchi Churna* (#80 sieve), *Guduchi Svarasa*, and *Guduchi Kwatha,* mature fresh medium sized diameter (1.6–2.0 cm) stems of *Guduchi,* climbing on neem (*Azadirachta indica*) tree were collected in monsoon season from the same vicinity (from non polluted, wild area of ‘Moti Panchasara’ village of Jamnagar district, Gujarat) to avoid any phyto-geographical differences. The physical impurities and outer exfoliating skin were removed and washed thoroughly with potable water. The collected plant material was authenticated (voucher specimen no- Phm/6,198) by the concerned authority in the Pharmacognosy laboratory of the institute.

Fresh *Guduchi* stems were taken to prepare different dosage forms as per classical Ayurvedic guidelines i.e., *Sadaiva ardra prayojyeta* (always use in fresh state) (Shastri, 2005; Sharma et al., 2012). *Guduchi* stems growing with the support of neem tree were selected, because it is believed to be the best as the synergy between these plants enhances its efficacy (Kinghorn, 2003; Bhalerao et al., 2012). Medium diameter or thumb sized stems from the same plant with uniform maturity was selected for study, as this is advocated to use for preparing *Guduchi-*based formulations (Acharya, 2008). Ayurvedic guidelines advocate collecting stems of medicinal plants in rainy/spring season. These ancient claims are supported by recent reports that the concentration or percentage of total alkaloids and the anti-diabetic biomarkers, tinosporaside and berberine of *Guduchi* are bit higher in rainy/monsoon season (Sharma et al., 2013c; Choudhry et al., 2014). Therefore the same harvesting time was selected for *Guduchi* collection.

**FIGURE 1:**
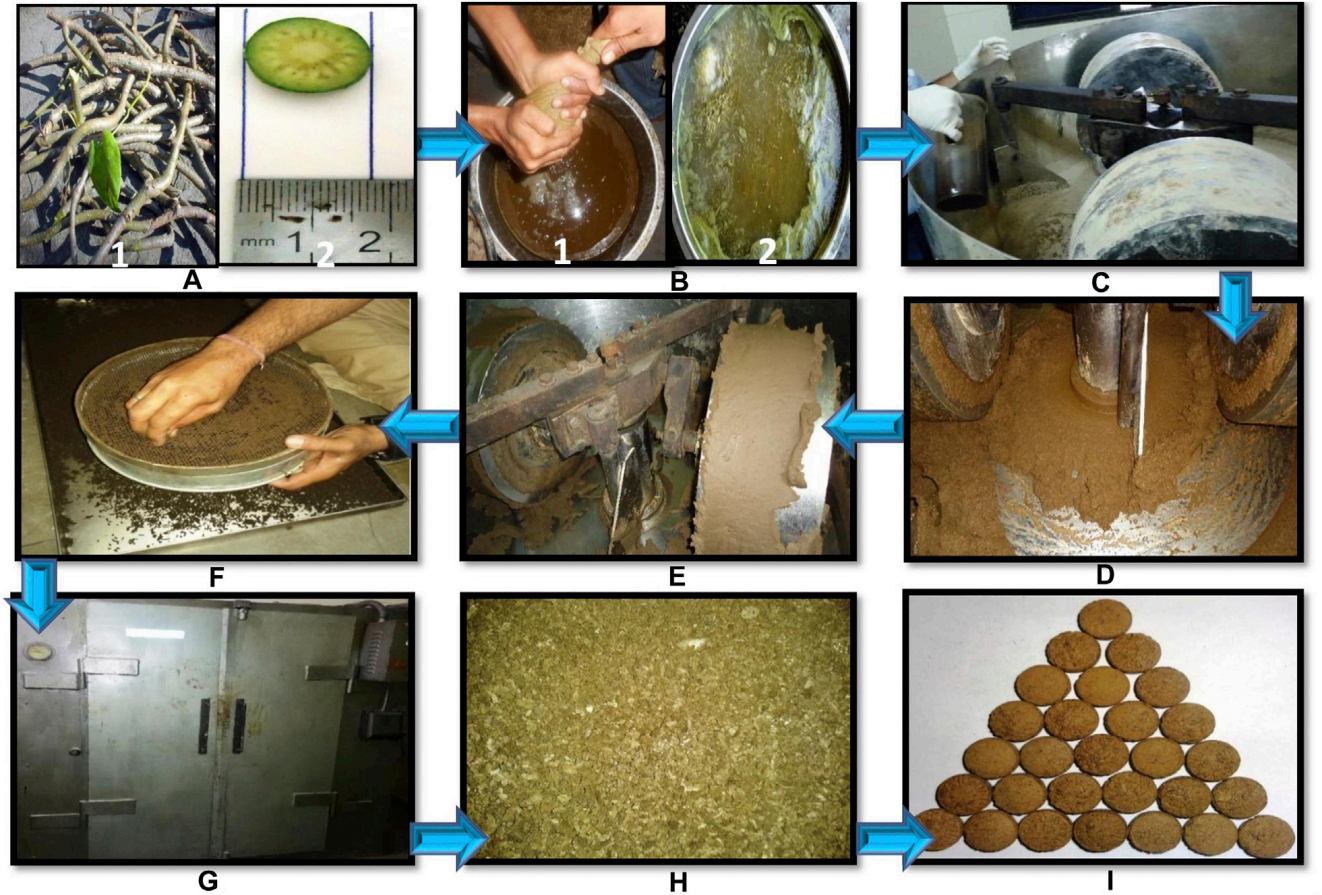
Unit operating process of Svarasa Bhavita Guduchi Churna (SBGC), Kwatha Bhavita Guduchi Churna (KBGC), and Jala Bhavita Guduchi Churna (JBGC) samples preparation by traditional Bhavana method. **(A)**- (1) fresh Guduchi stems (2) chopped/crushed, **(B)**- (1) expressed juice (for SBGC batch) (2) decoction was prepared (for KBGC batch), **(C)**- wet grinding of Guduchi Churma with liquid media (Guduchi juice for SBGC batch, Guduchi decoction for KBGC batch, and potable water for JBGC batch) in edge-runner, **(D)**- soft and fine mass formed during grinding, **(E)**- observation of Subhavita Lakshana (Confirmatory tests for completion of levigation), **(F)**- wet granulation, **(G)**- hot air over drying, **(H)**- dry granules, **(I)**- preparation of tables.

In pharmaceutical process, three different batches of SBGC, KBGC, and JBGC were prepared. GC was subjected to 07 times *Bhavana* separately in edge-runner with *Guduchi Svarasa* (fresh expressed juice of *Guduchi* stems) - for SBGC batch, *Guduchi Kwatha* (decoction of *Guduchi* stems) - for KBGC batch) and potable water - for JBGC batch. The whole unit operating process involved in the preparation of SBGC, KBGC, and JBGC is exemplified in Figure 1. The whole pharmaceutical process of 07 times *Bhavana* for each formulation SBGC, KBGC, and JBGC was completed in the same season therefore excluding possibility of major changes in environmental conditions. The duration of wet grinding for each cycle of *Bhavana* was kept constant to 9 h so as to maintain homogeneity in pharmaceutical processing among the batches of three formulations. The finished products were collected, weighed and their compressed tablets (500 mg each) were prepared and stored in air-tight sterile glass containers along with small pieces of cotton in them.

### *In-Vitro α*-Amylase and *α*-Glucosidase Inhibitory Studies

#### Chemicals and Reagents

Soluble starch (Merck), 3, 5-dinitrosalicylic acid (DNSA) (S D fine-chem Ltd.), phosphate buffer (0.02 M), hexane, dichloro methane (DCM) (S D fine-chem Ltd.), chloroform (S D fine-chem Ltd.), porcine pancreatic *α*-amylase (PPA) (Sigma-Aldrich) and acarbose (Bayer pharma) were used.

#### Test Materials

From the tablets of preparations, 5, 10, 15 and 25% of GC, JBGC, KBGC and SBGC solutions were prepared in different solvents (phosphate buffer, hexane, dichloro methane, chloroform).

#### α-Amylase Inhibitory Activity

The *α*-amylase inhibitory activity was determined according to the standard methods (Kumar et al., 2010; Sudha et al., 2011). 200µL of phosphate buffer was mixed with 200 µL of alpha-amylase solution and 500 µL of various concentrations of test samples (5–25%) separately, were incubated at room temperature for 15 min and followed by addition of 200 µL of starch solution (1%) in all test tubes and further incubated for 10 min at 37°C. The enzymatic reaction was stopped by adding 400 µL of freshly prepared DNS reagent and placing in boiling water bath for 5 min. The mixture was allowed to cool, diluted with 15 ml of distilled water and absorbance was determined at 540 nm (Double beam systonic spectrometer-2201). The control samples were also prepared accordingly without any plant extracts and were compared with the test samples containing various concentrations of the plant extracts prepared with different solvents. The results were expressed as % inhibition.

#### α-Glucosidase Inhibitory Activity

The *α*-glucosidase inhibitory activity was determined using the standard method (Chougale et al., 2009; Tripathi, 2014). The enzyme solution was prepared by dissolving 0.5 mg *α*-glucosidase in 10 ml phosphate buffer (pH 7.0) containing 20 mg bovine serum albumin. It was diluted further to 1:10 with phosphate buffer just before use. Test sample solutions (5,10,15, and 25%) were prepared and 5 μL each of the sample solutions or DMSO (sample blank) were then added to 250 μL of 20 mM *p*-nitrophenyl-α-d -glucopyranoside and 495 μL of 100 mM phosphate buffer (pH 7.0). It was pre-incubated at 37°C for 5 min and the reaction started by addition of 250 μL of the enzyme solution, after which it was incubated at 37°C for exactly 15 min 250 μL of phosphate buffer was added instead of enzyme for blank. The reaction was then stopped by addition of 1000 μL of 200 mM Na2CO3 solution and the amount of *p*-nitrophenol released was measured by reading the absorbance of sample against a sample blank (containing DMSO with no sample) at 400 nm using UV visible spectrophotometer. The results were expressed as % inhibition.

#### Data Analysis

Experimental data of *α*-amylase and *α*-glucosidase inhibitory activity was expressed by multivariate analysis, principal component analysis (PCA) with the help of Unscrambler software (Sharma et al., 2013c).

### *In Vivo* Anti-Hyperglycemic Studies

#### Experimental Animals

Swiss albino mice of either sex weighing 30 ± 5 g were obtained from animal house attached to Pharmacology laboratory of Institute for Postgraduate Teaching and Research in Ayurveda, Gujarat Ayurved University, Jamnagar, Gujarat, India, for experiments and maintained under standard experimental and husbandry conditions.

The animals were housed in each cage made of poly-propylene with stainless steel top grill. The dry wheat (post hulled) waste was used as bedding material and was changed every morning. The animals were exposed to 12 h light and 12 h dark cycle with the relative humidity of 50–70% and the ambient temperature during the period of experimentation was 22 ± 03°C. Animals were fed with Amrut brand rat pellet feed supplied by Pranav Agro Mills Pvt. Limited and drinking water *ad libitum*. The experiments were carried out after obtaining permission from Institutional Animal Ethics Committee (IAEC Approval number: IAEC/13/2013/01/PhD).

#### Dose Selection and Schedule

The clinical dose of *Bhavita Guduchi Churna* was taken based on available references as 4 g/day (Sharma et al., 2014c; Shingadiya et al., 2017). The dose for experimental study was calculated by extrapolating the human dose to animals (520 mg/kg) based on the body surface area ratio by referring to the tables of Paget and Barnes (Paget and Barnes, 1964). The test drugs were suspended in distilled water with suitable concentration and administered according to the body weight of the animals by oral route with the help of gastric oral cannula.

#### Instruments Used

One touch Glucometer (Lifeline Surgicals, New Delhi, India) with *Ez Smart* strips, weighing scale, disposable needle and syringe, mono pan balance and mortar and pestle.

#### Experimental Study

The hypoglycemic activity and anti-hyperglycemic activities were carried out by modifying previously described method of Pilkhwal et al. (2010). The selected mice were acclimatized for 7 days prior to experiments. The animals were divided randomly into relevant groups of six animals each. Then both activities were evaluated for the test drugs as per the following protocols:

#### Hypoglycaemic Activity

Swiss albino mice of either sex were randomly divided into five groups of six animals each. The first group received distilled water and served as water control group (10 ml/kg, po). Second group served as standard control group to which glibenclamide (0.65 mg/kg) was administered. Third, fourth and fifth groups were received JBGC, KBGC and SBGC respectively in the dose of 520 mg/kg, orally.

The animals were fasted overnight prior to the experiment and in the morning the fasting initial reading of blood sugar level (BSL) was measured with the help of One Touch Ez Smart CE0537 Glucometer, by using One Touch Ez Gluco test strips as per user’s guideline by collecting the blood sample from tail vein under aseptic conditions. Then the water, test drug and standard drug were administered to respective groups. The BSL was recorded after 1, 2, 3 and 5 h of test drug administration for assessing the hypoglycemic effect after drug administration.

#### Anti-Hyperglycaemic Activity

The selected animals were randomly divided into five groups of six animals each. First group served as glucose control group to which glucose solution (5 g/kg, po) was administered. Second group served as standard control group to which glibenclamide (0.65 mg/kg, po) was administered. Third, fourth and fifth groups were received JBGC, KBGC and SBGC respectively in the dose of 520 mg/kg, oral.

Animals were fasted overnight prior to the experiment and in the morning the fasting initial BSL was measured as mentioned in hypoglycemic activity. Test drugs and reference standard drug were given to the respective group of animals as per the body weight. After 1 h of drug administration, glucose (5 g/kg, po) solution was administered to all groups orally by dissolving it in distilled water. Thereafter BSL was recorded at 30, 60, 90 and 120 min of post glucose overload for accessing the anti-hyperglycemic activity of test drug (Malalavidhane et al., 2000; Sachdewa et al., 2001; Kingsley et al., 2013).

#### Statistical Analysis

Results were presented as Mean ± SEM, difference between the groups was statistically determined by Student paired ‘t’ test with initial values of respective groups and unpaired ‘t’ test with control group. The data also assessed through Anova followed by Dunnet’s multiple ‘t’ test. The level of significance set at *p* < 0.05. The level of significance was noted and interpreted accordingly.

#### High Performance Thin Layer Chromatography Studies

All chemicals utilized were of analytical grade and acquired from Merck Ltd. Berberine chloride hydrate, CAS number - 14,050–10G; purity 90% was purchased from Sigma Aldrich (St. Louis, MO, United States). 5 g of each GC, SBGC, KBGC and JBGC formulation was refluxed with 50 ml of methanol for around 2 h. The subsequent solutions were filtered and concentrated using a rotary evaporator. The resultant extracts i.e., 240 mg (GC); 137 mg (SBGC); 200 mg (KBJC) and 100 mg (JBJC) were transferred in to 50 ml volumetric flask. The standard Berberine chloride hydrate 10 mg was dissolved in 10 ml methanol and finally standard concentration 1000 µg/ml was made. The calibration curve prepared with different volumes of standard stock solution (1, 2, 3, 4, 5, 7 µL) were spotted on HPTLC plate (20 × 10 cm) for berberine chloride hydrate followed by spotting (3, 4, 2, 1.8 µL) GC, JBGC, SBGC and KBJC respectively sample stock solutions in triplicate. Samples were applied as bands 4 mm wide keeping 12-mm distance from the left edge using Camag Linomat V applicator with a 100 µL test syringe at a steady application rate of 150 nL s^−1^. The mobile phase used was n-hexane, ethyl acetate, glacial acetic acid and methanol (10:1.1:1.1:2.5, v/v). After development, the plates were dried and observed in CAMAG TLC Visualizer at 254 nm. The developed plate was then scanned at 254 nm using CAMAG TLC densitometric Scanner 3 incorporated with WinCATS 1.4.8 programming (Doshi et al., 2014; Kamboj and Saluja, 2017).

## Results

### α-Amylase Inhibitory Activity

**FIGURE 2:**
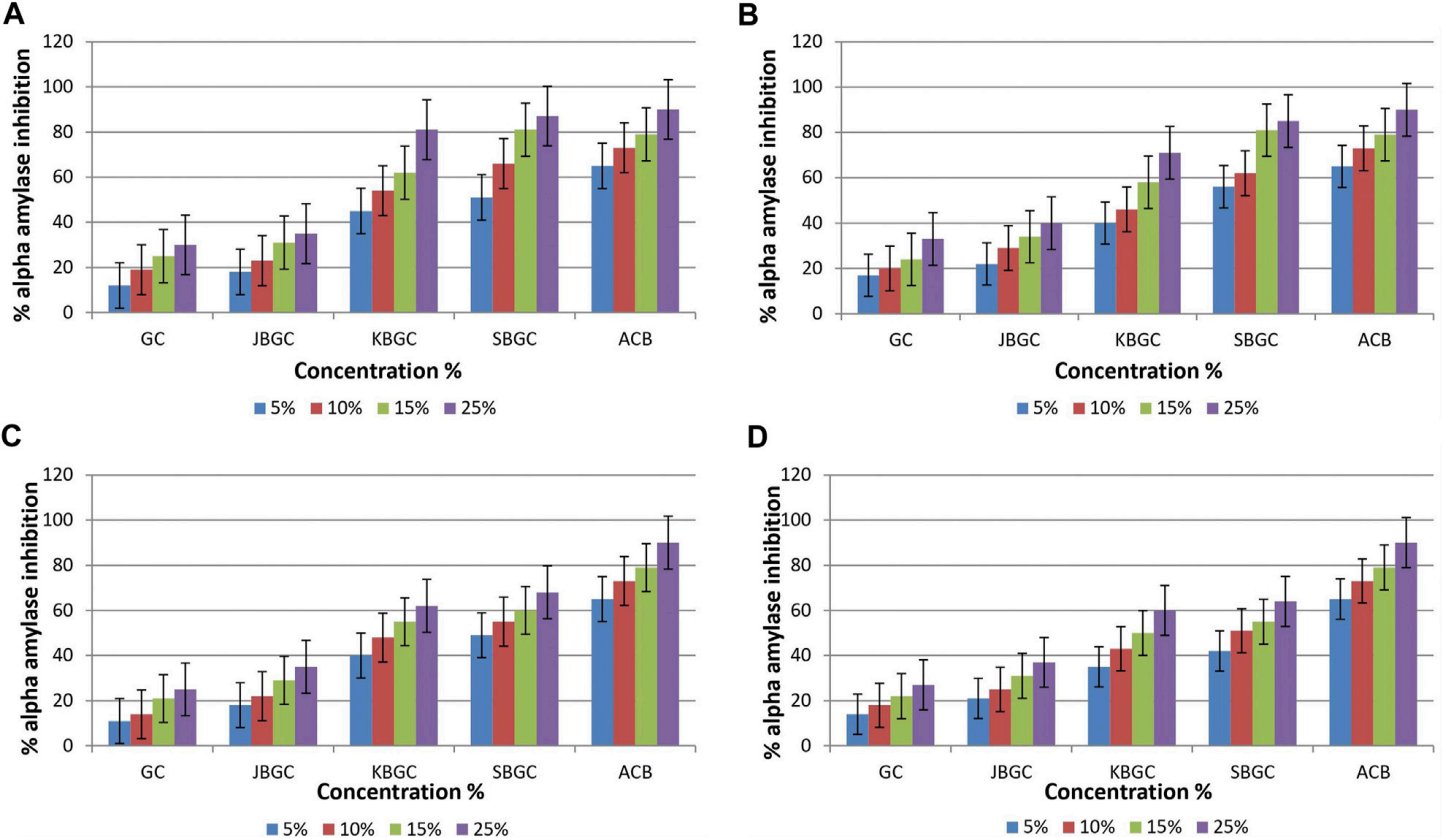
*α*-amylase inhibitory activity of *Guduchi Churna* (GC), *Svarasa Bhavita Guduchi Churna* (SBGC), *Kwatha Bhavita Guduchi Churna* (KBGC), and *Jala Bhavita Guduchi Churna* (JBGC). **(A)** In phosphate buffer solution (PBS) **(B)** In dichloro methane (DCM) **(C)** In chloroform **(D)** In hexane.

The test formulations in different solvents were studied for inhibitory activity of *α* - amylase enzyme (Figure 2). It is evident from graphs that maximum *α* - amylase inhibition was demonstrated by SBGC, followed by KBGC, JBGC and GC formulations in comparison to the standard acarbose. In phosphate buffer saline SBGC formulation showed maximum activity (87%) which is comparable with acarbose (90%).

### α-Glucosidase Inhibitory Activity

**FIGURE 3:**
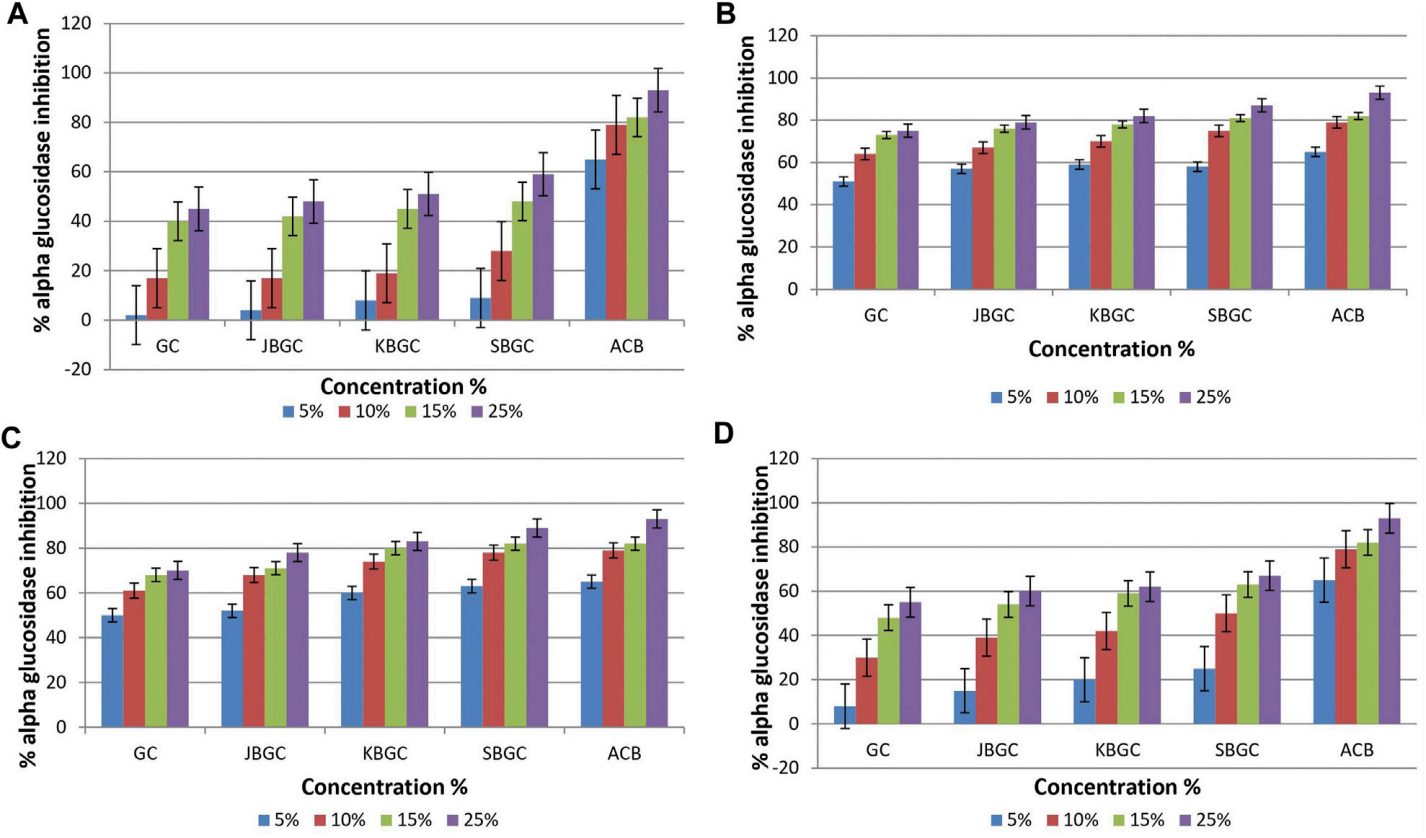
*α*-glucosidase inhibitory activity of *Guduchi Churna* (GC), *Svarasa Bhavita Guduchi Churna* (SBGC), *Kwatha Bhavita Guduchi Churna* (KBGC), and *Jala Bhavita Guduchi Churna* (JBGC). **(A)** In phosphate buffer solution (PBS) **(B)** In dichloro methane (DCM) **(C)** In chloroform **(D)** In hexane.

Data with respect to effect of GC, JBGC, KBGC and SBGC on *α*-glucosidase inhibitory activity are represented in Figure 3. It is evident from the graphs that maximum inhibition was demonstrated by DCM and chloroform extracts of SBGC, followed by KBGC, JBGC and GC in comparison to standard acarbose. Maximum inhibition (89%) was reported by SBGC formulation at 25% solution of DCM.

### Differences Among the High, Medium and Low *α*-Amylase Inhibitory Activity Groups

**FIGURE 4:**
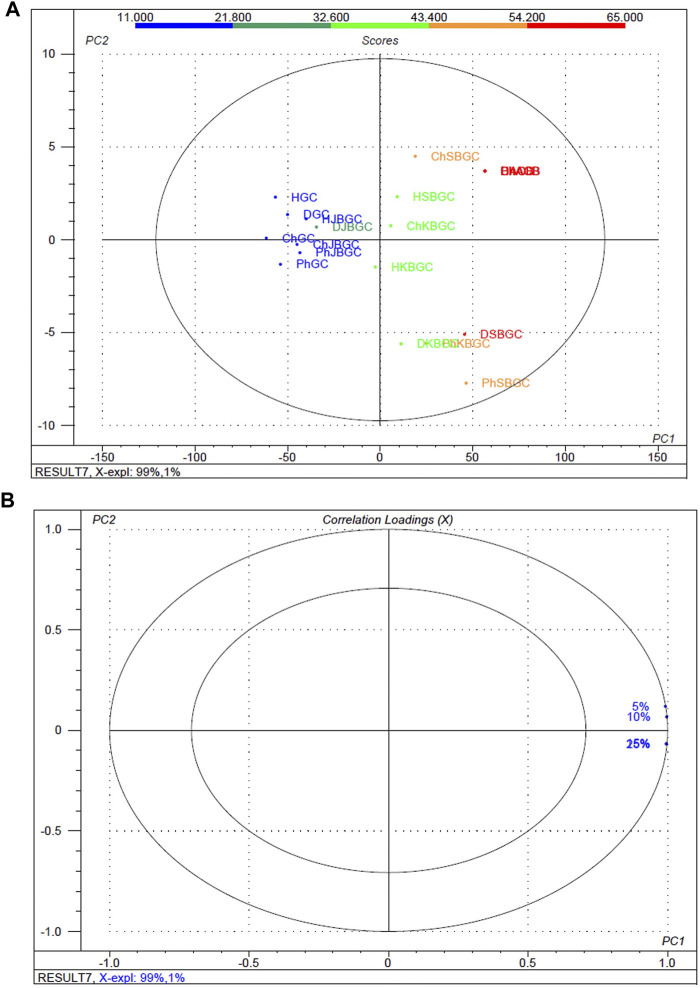
Multivariate statistical analysis of codified different polarity *Guduchi* samples (Ph: phosphate buffer solution, D: dichloro methane, Ch: chloroform, and H: hexane) based on an unsupervized PCA model. **(A)** Unsupervized PCA score plot **(B)** Loading plot of *α*-amylase inhibitory activity of GC, JBGC, KBGC, SBGC formulations and various concentrations.

To better screen the fuzziness and integrity of the various concentration and polarity codified *Guduchi* samples differentiating their *α*-amylase inhibitory activity (Figure 4), in the experimental samples, % of inhibitory outcome were separated five groups, high (≤54%), medium (≤43%) and very low (≥21%). An overview of the differences in the respective concentrations (5, 10, 15 and 25%) samples was obtained using unsupervized PCA, which takes into account all variables (Figures 4A, Figures 4B). The 1st and 2nd principal components explained 91 and 1% of the total variance respectively. In score plot, we can observe clear separation between SBGC and KBGC, while there was not clear separation of GC and JBGC because of *α*-amylase activity as a continuous variable. The SBGC samples have high content of active marker compounds may have great potential for preventing postprandial hyperglycemia followed by KBGC, JBGC and GC medicaments in comparison to standard acarbose.

### Differences Among the High, Medium and Low *α*-Glucosidase Inhibitory Activity

**FIGURE 5:**
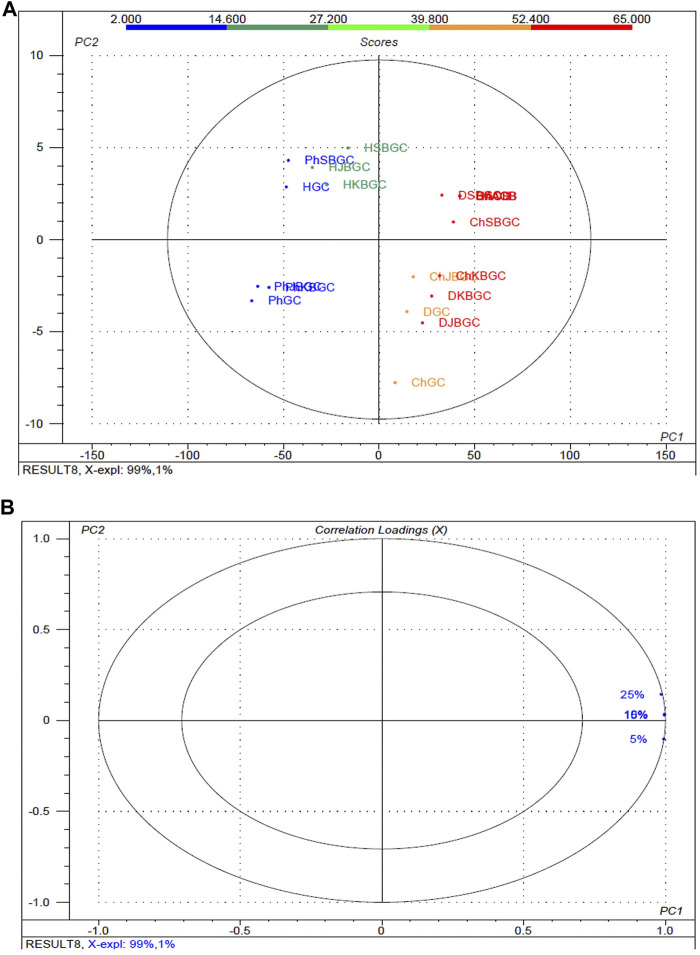
Multivariate statistical analysis of codified different polarity *Guduchi* samples (Ph: phosphate buffer solution, D: dichloro methane, Ch: chloroform, and H: hexane) based on an unsupervized PCA model. **(A)** Unsupervized PCA score plot and **(B)** Loading plot of *α*-glucosidase activity of GC, JBGC, KBGC, SBGC formulations and various concentrations. The ellipse represents the Hotelling T_2_ with 95% confidence.

The experimental findings of the various concentration and polarity codified *Guduchi* samples differentiating their *α*-glucosidase inhibitory activity (Figure 5), in the experimental samples, % of inhibitory outcome were separated five groups, high (≤52.4%), medium (≤39%) and very low (≥14%). An overview of the differences in the respective concentrations (5, 10, 15 and 25%) samples was obtained using unsupervized PCA, which takes into account all variables (Figures 5A, Figures 5B). The first and 2nd principal components explained 99 and 1% of the total variance respectively. In score plot, we can observe clear separation of between GC and KBGC, while there was not clear separation of SBGC and JBGC because of *α*-glucosidase activity as a continuous variable. Furthermore, DCM and chloroform extract of SBGC with a high amount of active marker compounds may have more synergy and supra additive therapeutic effect, followed by KBJC, JBGC and GC in comparison to standard acarbose.

### Hypoglycemic Activity

**FIGURE 6:**
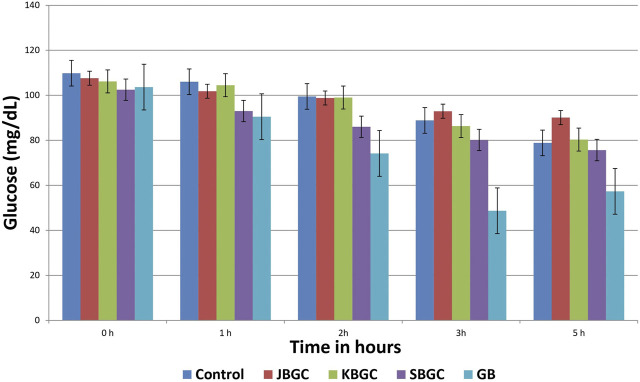
Effect of *Svarasa Bhavita Guduchi Churna* (SBGC), *Kwatha Bhavita Guduchi Churna* (KBGC), and *Jala Bhavita Guduchi Churna* (JBGC) on BSL in normal fasted mice.

Table 1 and Figure 6 represent the results of SBGC, KBGC, JBGC formulations on glucose concentration in normal fasted mice. All test formulations has produced a marginal hypoglycemia during the experimental studies. However the glucose concentrations were restored to that of pretreatment level (0 h). GB treated group leads to significant decrease in blood glucose level at almost all the time intervals in comparison to control group and maximum protection was observed at 3^rd^ hour with 45.20%.

**TABLE 1:** Effect of JBGC, KBGC and SBGC on blood sugar level in normal overnight fasted Swiss albino mice at various time intervals.

Blood glucose level (mg/dl)					
Groups	Initial (mg/dl)	1 h (mg/dl)	2 h (mg/dl)	3 h (mg/dl)	5 h (mg/dl)
**WC**	109.83 ± 2.24	106.00 ± 2.80	99.50 ± 1.70*	88.83 ± 1.90*	78.83 ± 1.85*
**JBGC**	107.60 ± 1.32	101.80 ± 1.49	98.80 ± 4.85*	92.92 ± 2.65**	90.10 ± 2.39*
		(4.12)	(0.7)		
**KBGC**	106.16 ± 3.05	104.50 ± 1.46*	99.00 ± 3.59*	86.33 ± 4.01*	80.33 ± 2.42*
		(1.41)	(0.5)	(2.81)	
**SBGC**	102.50 ± 1.01	93.00 ± 3.51**	86.00 ± 5.46*	80.16 ± 3.92*	75.66 ± 4.520*
		(12.26)	(13.56)	(9.76)	(4.02)
**GB**	103.64 ± 4.82	90.5 ± 2.32*	74.17 ± 1.25*	48.67 ± 2.26*	57.33 ± 4.84**
		(14.62)	(25.45)	(45.20)	(27.27)

WC, Water control; JBGC, Jala Bhavita Guduchi Churna; KBGC, Kwatha Bhavita Guduchi Churna; SBGC, Svarasa Bhavita Guduchi Churna; GB, Glibenclamide as standard control. *p < 0.05, **p < 0.01 are in comparison with initial blood glucose levels of the mice (0 h) in the respective group. Figures in parenthesis indicate the percentage decrease.

Values are mean ± S.E.M; *n* = 6.

### Anti-Hyperglycemic Activity

**FIGURE 7:**
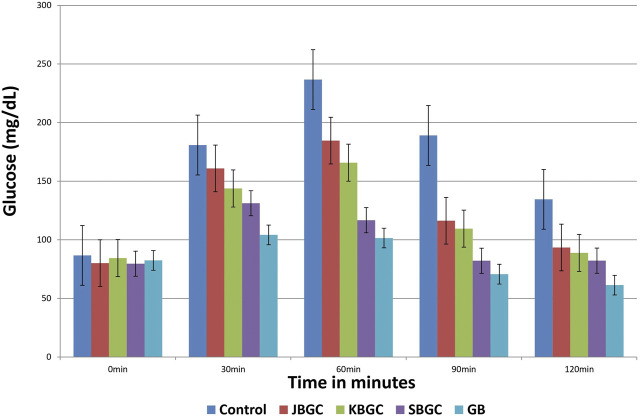
Effect of *Svarasa Bhavita Guduchi Churna* (SBGC), *Kwatha Bhavita Guduchi Churna* (KBGC), and *Jala Bhavita Guduchi Churna* (JBGC) on BSL in glucose overloaded mice.

The effects of JBGC, KBGC and SBGC formulations in glucose tolerance test in mice are presented in Table 2 and Figure 7. All formulations are shown significant activity at an oral dose of 520 mg/kg. The maximum protection was observed at 90 min after glucose load. Among all formulations SBGC and KBGC were produced maximum protection at 90 min. Glibenclamide was used as standard and produced maximum protection at 90 min with percentage protection of 62.60%. The maximum glucose concentration was observed at 60 min with glucose concentration of 236.6 mg/dl. The percentage protection at 90 min produced by JBGC, KBGC and SBGC is 38.46, 42.06 and 56.56 respectively when tested at dose of 520 mg/kg, these results are comparable in activity produced by glibenclamide at a dose of 0.65 mg/kg. SBGC produced significant anti-hyperglycemic effect at all-time intervals in comparison to control group. Overall, SBGC produced pronounced anti-hyperglycemic effect followed by KBGC and JBGC.

**TABLE 2:** Effect of JBGC, KBGC and SBGC on blood sugar level in glucose overloaded Swiss albino mice at various time intervals.

Blood glucose level (mg/dl)					
Groups	0 min	30 min	60 min	90 min	120 min
**GC**	86.66 ± 2.84**	180.83 ± 3.84**	236.6 ± 4.49**	189.0 ± 2.3**	134.5 ± 2.89**
**JBGC**	80.1 ± 1.62**	160.9 ± 2.89**	184.6 ± 2.62**	116.3 ± 1.26**	93.4 ± 3.42**
		(11.11)	(21.99)	(38.46)	(30.55)
**KBGC**	84.4 ± 3.97**	143.8 ± 4.56**	165.8 ± 3.12**	109.5 ± 3.15**	88.8 ± 1.94**
		(20.47)	(29.94)	(42.06)	(33.97)
**SBGC**	79.6 ± 4.58**	131.2 ± 1.97**	116.7 ± 4.23**	82.1 ± 1.59**	82.2 ± 3.04**
		(27.44)	(50.67)	(56.56)	(38.88)
**GB**	82.42 ± 5.12**	104.17 ± 2.68**	101.5 ± 3.58**	70.67 ± 1.36**	61.33 ± 2.33**
		(42.39)	(57.10)	(62.60)	(54.40)

GC, Glucose control; JBGC, Jala Bhavita Guduchi Churna; KBGC, Kwatha Bhavita Guduchi Churna; SBGC, Svarasa Bhavita Guduchi Churna; GB, Glibenclamide as standard control. *p < 0.05, **p < 0.01 are in comparison with initial blood glucose levels of the mice (0 h) in the respective group. Figures in parenthesis indicate the percentage decrease.

Values are mean ± S.E.M; n = 6.

### High Performance Thin Layer Chromatography Studies

**FIGURE 8:**
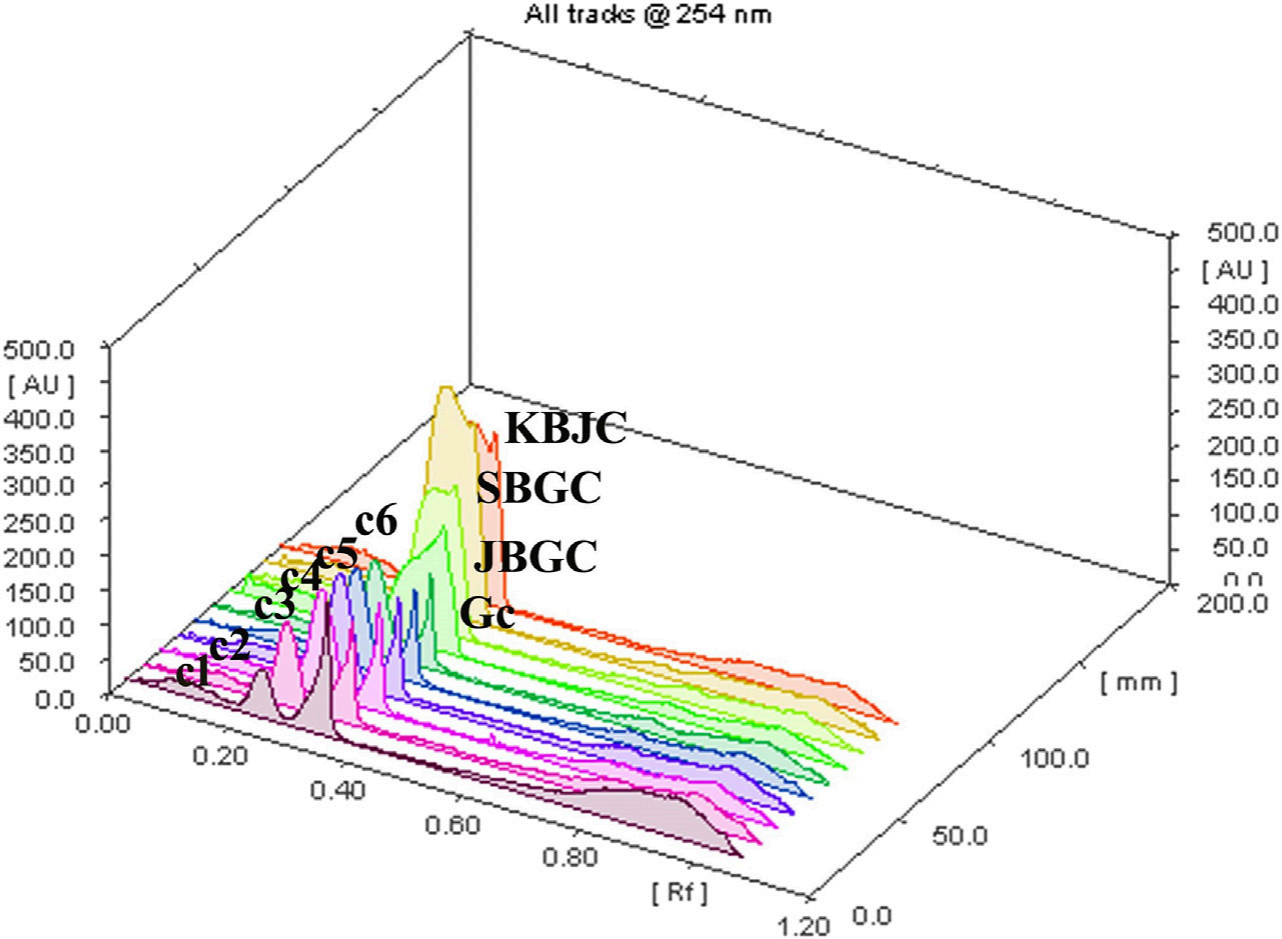
TLC plate scanned at 254 nm showing berberine chloride hydrate in the 3D samples chromatogram.

The developed mobile phase consisting of n-hexane, ethyl acetate and methanol (10:1.1:1.1:2.5, v/v) gave better, sharp and well defined peak resolution for standard (berberine chloride hydrate-a well-known anti-diabetic compound) as well as a test samples. The developed HPTLC method resolved the standard compound at R_F_ value of nearly about 0.28 ± 0.02 for berberine chloride hydrate for confirming the presence in coded extract visualized by band parallel to standard spot along with other resolved components in the developed TLC plate. The TLC plates were scanned at 254 nm and to identify of berberine chloride hydrate in the sample chromatogram was confirmed by three-dimensional (3D) chromatogram (Figure 8) obtained after densitometric scanning. The calibration curve was linear range of (200–1,400 ng/spot) for berberine chloride hydrate. The linear regression of the berberine chloride hydrate standard was determined with *R* ^2^ ± SD = 0.991 ± 3.21% with regression line; y = 7.336x + 459.8. The quantification of berberine chloride hydrate in the respective codified samples is depicted in Table 3. The peak corresponding to berberine chloride hydrate and berberine from the codified samples solution had the same retardation factor (R_F=_0.28 ± 0.02).

**TABLE 3:** The berberine chloride hydrate present in different codified samples.

Samples (coded methanolic extract)	Conc^n^ prepared (mg/ml)	Spotting volume (µL)	Berberine chloride hydrate determined (ng/spot)	Extractive value (%)	% Marker in extract	% Of marker in raw powder (g)
**GC**	2.74	3	963.44	2.74	11.72	0.32
**JBGC**	2	4	2024.63	2	25.30	0.51
**SBGC**	4	2	2,563.14	4	32	1.28
**KBGC**	4.8	1.8	1,222.23	4.8	14.14	0.59

GC, Guduchi Churna; JBGC, Jala Bhavita Guduchi Churna; KBGC, Kwatha Bhavita Guduchi Churna; SBGC, Svarasa Bhavita Guduchi Churna; GB, Glibenclamide as standard control.

## Discussion

This study was carried out not only to validate the traditional uses of *Guduchi* and its preparations in diabetes (Sharma et al., 2014b) but also to initiate search for newer pharmacophores with specificity toward pancreatic *α*-amylase or *α*-glucosidase. Structurally as well as mechanistically, PPA is closely related to HPA (human pancreatic *α*-amylase) (Brayer et al., 1995). Hence, sequential solvent extracts of different formulations of *Guduchi* viz. GC, JBGC, KBGC and SBGC were screened for the presence of PPA inhibitors, the lead extracts quantified for PPA inhibition under *in-vitro* conditions. Primary screening for *α*-amylase inhibition was performed based on starch-iodine color complex formation. Different extracts of GC, JBGC, KBGC and SBGC were prepared in various solvents such as phosphate buffer, DCM, chloroform and hexane extracts. All these extracts were screened, and all of them tested positive for PPA inhibition by chromogenic DNSA method. It was noted that phosphate buffer and DCM extracts of SBGC exhibited strong PPA inhibitory potential (>80% inhibition, in 15 and 25% concentrations) followed by KBGC, JBGC and GC. In *α*-glucosidase inhibitory activity, maximum inhibition was shown by DCM and chloroform extracts of SBGC (>85% inhibition, in 15 and 25% concentrations), followed by KBGC (>80% inhibition, in 15 and 25% concentrations), JBGC and GC.

In both *α*-amylase and *α*-glucosidase inhibitory activities, a dose/concentration dependent effect was observed on increasing the concentrations of the extract solution, suggesting a competitive type of inhibition. Our results suggests that extracts of GC, JBGC, KBGC and SBGC act effectively as PPA inhibitors leading to a reduction in starch hydrolysis and hence eventually to lowered glucose levels. Also, a good discrimination among respective codified samples is easily obtained from PCA analysis, may be due to supra-additive active metabolite load in context of *α*-amylase and *α*-glucosidase inhibitory activity. Our study shows that SBGC followed by KBGC were effectively inhibiting *α*-amylase and *α*-glucosidase enzyme and therefore it may be used as hypoglycemic agents in the management of post prandial hyperglycemia. The study suggests that one of the targets for hypoglycaemic property of *Guduchi* is *α*-amylase and *α*-glucosidase inhibition, where SBGC demonstrated a highly promising and effective strategy for diabetes, which may serve as a lead for isolation and characterization of compounds responsible for it.

Taking leads from encouraging enzyme inhibitory results, *in-vivo* experiments were carried out to understand the effects of extracts on glycemic levels of experimental animals. An ideal antihyperglycemic agent should decrease the elevated blood glucose levels and should maintain the normal blood glucose levels (Venkatesh et al., 2003). The decrease in blood glucose levels below the normal i.e., hypoglycemia, is the major side effect of insulin and oral hypoglycemic agents. In the present investigation, all extracts were tested for their hypoglycemic activity and the results showed partial hypoglycemia in normal mice, where as glibenclamide showed maximum protection (45.20%.) at 3rd hour.

Glucose tolerance test is a preliminary method to assess the ability of drug to reduce the increased blood glucose levels or not (Kingsley et al., 2013). In this screening method, the animals are loaded with challenging dose of glucose (5 g/kg) after 60 min of test, standard drug administration. After glucose load, the raise in glucose concentration is observed from 30 min and reached maximum at 60 min and decreased by 120 min. SBGC, KBGC, and JBGC extracts produced significant activity at 90 min, however, the maximum significant (*p* < 0.01) activity was produced at 520 mg/kg by SBGC, KBGC, and JBGC is 56.56, 42.06 and 38.46 respectively. SBGC produced significant anti-hyperglycemic effect at all-time intervals in comparison to control group. Overall, SBGC produced pronounced anti-hyperglycemic effect followed by KBGC and JBGC. The standard glibenclamide (10 mg/kg) produced a significant maximum protection at 90 min (62.60%). The observed promising results of the test drugs on glycemic levels could be due to the increased concentration of the anti-diabetic phytoconstituents during *Bhavana* process. It also has been reported that the anti-diabetic activity of this plant is primarily due to improving the entry of glucose into the peripheral tissues and organs like the liver and decreasing the activity of phosphorylase in the liver, thereby it may prevent the release of glucose into the blood (Puranik et al., 2010). The same mechanism may be involved in the observed activity profile; however further detailed studies are needed to understand the exact pharmacodynamics involved.

In view of the potential anti-diabetic importance of *Guduchi*, and promising results obtained in present study from *in-vitro* and *in-vivo* investigations, an attempt has been made to chromatographically quantify the berberine level in *Bhavita Guduchi* samples (SBGC, KBGC, and JBGC). The isoquinoline alkaloid ‘berberine’ has been tested and used successfully in experimental and human diabetes. It is reported to exhibit significant antioxidant and anti-hyperglycemic activity, inhibits FOXO 1 (Forkhead Box O1 Protein), which integrates insulin signaling with mitochondrial function, and activates AMPK (AMP-activated protein kinase), thus decreases the levels of blood sugar, cholesterol and maintains the blood pressure (Sharma and Batra, 2013). Berberine salts are also reported as bioavailabity enhancer (Cui et al., 2019). On the flip side, isolation of another anti-diabetic compound Tinosporaside, an 18-norclerodane glucoside*,* is a tedious and more reductionist way to sample cleanup (Puratchimani and Jha, 2007), which would be a distoration of Ayurvedic holistic principle. In the present study, HPTLC method was developed for the determination of berberine chloride hydrate in *Guduchi* formulations, which showed the presence of berberine chloride hydrate in SBGC (32%) > KBGC (25.3%) > JBGC (14.14%) > GC (11.72%) respectively in increasing way. In HPTLC, increased berberine level in *Bhavita* samples (in comparison to GC) signifies the role of *Bhavana* in increasing the concentration of phyto-constituents. The obtained HPTLC results also corroborate with the findings of *in-vitro* and *in-vivo* experiments, wherein *Bhavita* samples exhibited better results than crude *Guduchi Churna*, and specifically SBGC showed better activity profile among all other samples. The increased berberine level after *Bhavana* could be having the role as bioavailability enhancer or imparting a supra-additive effect. Therefore further investigations are required to understand the kinetic chemistry of *Bhavana* in adding of myriad of bioactive phytoconstituents or active metabolites during wet grinding process and its possible role in improving the absorption and bioavailability of drugs.

In Ayurvedic classics, only *Svarasa* (extracted juice) dosage form of *Guduchi* is mentioned to be used for diabetes and in Ayurvedic pharmaceutics the *Svarasa* dosage form is said to be more potent than *Kwatha* (decoction) (Sharma et al., 2014b). Also, the *Svarasa* is extracted whole juice that may have more extractive principles of the plant, while decoction is only aqueous soluble extract of the botanical. However owing to very short shelf life of *Svarasa* (3 h) (Gupta et al., 2011), other dosage forms of *Guduchi* (viz. *Churna* or *Kwatha*) are popular among traditional practitioners. Thus better activity profile of SBGC in present *in-vitro* and *in-vivo* experiments validates and substantiate the Ayurvedic claims to use *Guduchi Svarasa* in diabetes; and to prepare the *Svarasa Bhavita* dosage form appears to be an effective way to preserve the properties of *Svarasa*. Hence further shelf-life studies are required for better understanding.

Present findings endorse the use of these formulations of *Guduchi* to manage glycemic levels in type 2 diabetes management. Further *in-vivo* antidiabetic as well as clinical studies are warranted to substantiate these findings. More extensive works are also needed to explore these formulations for their antioxidant, cytoprotective, and immunomodulatory roles in the management of other pathological metabolic cascades involved in diabetes.

## Conclusion

In this study, better *α*-amylase, *α*-glucosidase inhibitory activities and significant anti-hyperglycemic effect of SBGC and KBGC ascertain definite role of *Bhavana* in augmentation of bioefficacy of drugs and suggest promising potential of these *Guduchi* formulations for the management of type 2 diabetes. This corroborate with findings of HPTLC study, wherein the percentage of anti-diabetic compound ‘berberine’ was found increased in *Bhavita* samples (maximum in SBGC, followed by KBGC and JBGC). The obtained results provide new leads to researchers to investigate these formulations apropos their pharmacokinetic and pharmacodynamic mechanistic roles, bioactivities on other therapeutic parameters as well as at clinical levels. This traditional Ayurvedic pharmaceutical concept of *Bhavana* can be utilized further in invention of new chemical moieties in the field of drug discovery and development.

## Data Availability

The original contributions presented in the study are included in the article/Supplementary Material, further inquiries can be directed to the corresponding author.
